# The Role of EREG/EGFR Pathway in Tumor Progression

**DOI:** 10.3390/ijms222312828

**Published:** 2021-11-27

**Authors:** Wan-Li Cheng, Po-Hao Feng, Kang-Yun Lee, Kuan-Yuan Chen, Wei-Lun Sun, Nguyen Van Hiep, Ching-Shan Luo, Sheng-Ming Wu

**Affiliations:** 1 Division of Cardiovascular Surgery, Department of Surgery, Wan Fang Hospital, Taipei Medical University, Taipei 11696, Taiwan; wanlicheng80@gmail.com; 2 Division of Cardiovascular Surgery, Department of Surgery, School of Medicine, College of Medicine, Taipei Medical University, Taipei 11031, Taiwan; 3 Division of Pulmonary Medicine, Department of Internal Medicine, Shuang Ho Hospital, Taipei Medical University, New Taipei City 23561, Taiwan; pohao.feng@gmail.com (P.-H.F.); lee4949@ms41.hinet.net (K.-Y.L.); 14388@s.tmu.edu.tw (K.-Y.C.); brontesun@gmail.com (W.-L.S.); nguyenhiep0320@gmail.com (N.V.H.); gberg7209@gmail.com (C.-S.L.); 4 Division of Pulmonary Medicine, Department of Internal Medicine, School of Medicine, College of Medicine, Taipei Medical University, Taipei 11031, Taiwan; 5 Graduate Institute of Clinical Medicine, College of Medicine, Taipei Medical University, Taipei 11031, Taiwan; 6 International PhD Program in Medicine, College of Medicine, Taipei Medical University, Taipei 11031, Taiwan

**Keywords:** epidermal growth factor receptor (EGFR), epiregulin (EREG), tumor microenvironment, cancer therapy

## Abstract

Aberrant activation of the epidermal growth factor receptor (EGFR/ERBB1) by erythroblastic leukemia viral oncogene homolog (ERBB) ligands contributes to various tumor malignancies, including lung cancer and colorectal cancer (CRC). Epiregulin (EREG) is one of the EGFR ligands and is low expressed in most normal tissues. Elevated EREG in various cancers mainly activates EGFR signaling pathways and promotes cancer progression. Notably, a higher EREG expression level in CRC with wild-type Kirsten rat sarcoma viral oncogene homolog (KRAS) is related to better efficacy of therapeutic treatment. By contrast, the resistance of anti-EGFR therapy in CRC was driven by low EREG expression, aberrant genetic mutation and signal pathway alterations. Additionally, EREG overexpression in non-small cell lung cancer (NSCLC) is anticipated to be a therapeutic target for EGFR-tyrosine kinase inhibitor (EGFR-TKI). However, recent findings indicate that EREG derived from macrophages promotes NSCLC cell resistance to EGFR-TKI treatment. The emerging events of EREG-mediated tumor promotion signals are generated by autocrine and paracrine loops that arise from tumor epithelial cells, fibroblasts, and macrophages in the tumor microenvironment (TME). The TME is a crucial element for the development of various cancer types and drug resistance. The regulation of EREG/EGFR pathways depends on distinct oncogenic driver mutations and cell contexts that allows specific pharmacological targeting alone or combinational treatment for tailored therapy. Novel strategies targeting EREG/EGFR, tumor-associated macrophages, and alternative activation oncoproteins are under development or undergoing clinical trials. In this review, we summarize the clinical outcomes of EREG expression and the interaction of this ligand in the TME. The EREG/EGFR pathway may be a potential target and may be combined with other driver mutation targets to combat specific cancers.

## 1. Introduction

### Erythroblastic Leukemia Viral Oncogene Homolog Signaling

**Figure 1:**
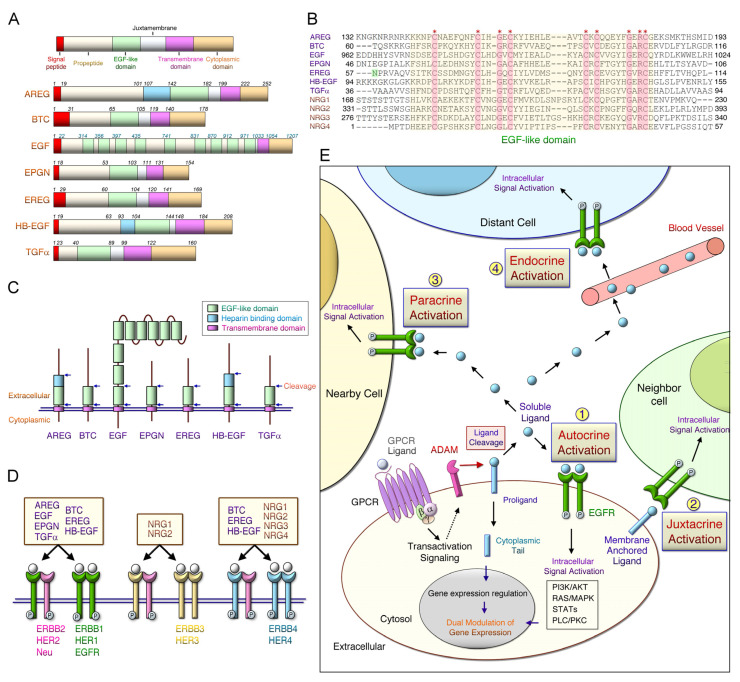
Protein domains, corresponding receptors of ERBB ligands and the possible activation pathways. (**A**) Erythroblastic leukemia viral oncogene homolog (ERBB) ligands include a signal peptide, a propeptide region, an epidermal growth factor (EGF)-like domain, a juxtamembrane, a transmembrane, and a cytoplasmic tail. Schematic representation of the membrane-anchored precursor form of the seven human EGF receptor (EGFR) ligands: EGF, transforming growth factor-a (TGFA), heparin-binding EGF-like growth factor (HB-EGF), amphiregulin (AREG), betacellulin (BTC), epiregulin (EREG), and epigen (EPGN). Amino acid residues that constitute the domains in the individual EGFR ligands are listed. EGF consists of nine EGF-like repeats. (**B**) The yellow region includes aligned amino acid sequences of EGF-like domains in seven EGFR ligands and neuregulin 1-4 (NRG1-4). Asterisks (*) indicate strictly conserved residues. The domains I and III EGFR interacted with the N57 residue of EREG. (**C**) Arrowheads indicate proximal and distal sites of cleavage in the EGF-like domains, which release to soluble ligands. (**D**) The ligand binds to the ERBB receptor to form receptor homodimers and heterodimers, and activates the intrinsic kinase domain that recruits proteins to activate intracellular signaling pathways. (**E**) Soluble ERBB ligands can bind to and activate their receptors (such as EGFR) through endocrine (distant cells), paracrine (adjacent cells), or autocrine (same cell) ways.

The expression of the erythroblastic leukemia viral oncogene homolog (ERBB) family is closely linked to tumor progression through the constitutive activation of downstream signaling, such as the epidermal growth factor (EGF) receptor (EGFR) pathway or through a somatic mutation; ERBB expression is enhanced during tumor microenvironment (TME) formation, cancer progression, and drug-resistance [1]. The ERBB family comprises transmembrane receptor tyrosine kinases, including the ERBB1/ EGFR)/HER (human EGF receptor) 1, ERBB2/HER2/Neu, ERBB3/HER3, and ERBB4/HER4 [2]. Activated ERBB mediates various signaling pathways including the RAS (rat sarcoma)/RAF (rapidly accelerated fibrosarcoma), phosphatidylinositol-4,5-bisphosphate 3-kinase (PI3K)/AKT (a serine/threonine protein kinase), phospholipase C (PLC)-γ1, and signal transducer and activator of transcription (STAT) pathways [3]. Several ligands can bind to EGFR, including EGF, epigen, transforming growth factor (TGF)-α, amphiregulin (AREG), epiregulin (EREG), betacellulin (BTC), and heparin-binding EGF (HB-EGF) (Figure 1). The simplified representation of these ligands with distinct functional domains are shown in Figure 1. The transmembrane EGFR ligands comprise an N-terminal signal peptide, pro-peptide region, the EGF-lik short juxtamembrane stalk, a hydrophobic transmembrane domain, and a cytoplasmic domain (Figure 1A). Neuregulins (NRGs) are a family containing the EGF-like domain proteins; they play an essential role in the development of the adult brain [4] and various cancers [5,6,7]. The most studied NRGs, such as the *NRG1* gene, produce six different types and 33 spliced isoforms, due to different transcriptional initiation sites and alternative splicing [4]. NRG proteins mainly contain EGF-like and transmembrane domains; however, the type-specific N-terminal region (type I, II, and IV-VI NRG1), an immunoglobulin-like domain, and the glycosylation site are dependent on the isoform [8,9]. Additionally, the identity of the overall protein sequence in these ligands is low [10] and a conserved EGF module including six cysteines is arranged as three disulfide bridges (Figure 1B). The spacing of EGF-motif in seven EGFR ligands can be represented as in the cysteines pattern CX_7_CX_4–5_CX_10_CXCX_8_C (X can be any amino acid). Notably, recent studies demonstrated that the N57 residue of EREG is pivotal for the interaction with the domains I and III of EGFR [11]. EGF is unique in that there are nine EGF motifs, although only the one adjacent to the cell membrane has the function of the EGFR binding domain (Figure 1C). EREG and HB-EGF contain an additional heparin-binding domain. The functional EGF module is located within approximately 25 residues of the transmembrane domain. The presence and spacing of additional specific residues further distinguishes EGFR ligands from NRGs containing EGF modules at the structural level, and defines high-affinity binding to EGFR [12]. Moreover, NRG1 and 2 selectively bind to ERBB3 (Figure 1D). Ligands such as BTC, HB-EGF, EREG, and NRG1-4 interact with the ERBB4. ERBB ligands bind to the extracellular domain of ERBB1, ERBB3, and ERBB4 receptors to form active homodimers or heterodimers. However, ligands do not directly bind to ERBB2 in the ligand-activated state, favoring homodimerization. In addition, ERBB2 proteins can be activated through interaction with other ERBBs. G-protein-coupled receptors (GPCRs) stimulate specific metalloproteinases, such as disintegrin and metalloproteinase (ADAM) family members, resulting in EGFR pro-ligand cleavage and transactive EGFR downstream cascade [13] (Figure 1E). Ectodomain shedding arises in diverse physiological responses, and the cleavage efficiency is mainly determined by the specific sequence in the cleavage site and the length of the membrane-proximal domain [14]. Soluble ligands bind to receptors, activating intracellular signaling on the original cell, neighboring cells, and distant cells through autocrine, paracrine (or juxtacrine), and endocrine pathways, respectively [12]. EGFR-medaited signaling pathways can be activated by binding to soluble ligands or membrane-anchored ligands by the juxtacrine pathway. In addition to the actions of soluble ligands, the free cytoplasmic tail (CT) of these ligands (e.g., pro-AREG CT) are required for basolateral sorting [15] and pro-HBE-GF CT can directly regulate gene expression [16]. The efficacy and specificity of intracellular signaling pathways are regulated by specific ligands, receptor dimerization, and interacting proteins that bind to the phosphorylated domains of ERBB [17].

In this review, we describe the signaling action of EREG, which plays a key role in cancer progression. Due to the complexity in the tumor development and heterogeneous cells in the TME, EREG expression and its action in distinct types of cancer cells accompanied with different genetic contexts, which confers different responses with drug treatments.

## 2. Critical Role of EREG in Early Tumor Development

### 2.1. The Normal Biological Functions of EREG Signaling

The EREG gene is located on human chromosome 4q13.3 and has a 24–50% sequence similarity with other members of the EGF family [18]. EREG gene expression is transcriptionally regulated by insulin [19], Sp1, AP-1, NF-κB [20], and AP-2 [21]. EREG is initially expressed as a transmembrane proform proteolytically cleaved by ADAM17 to release a soluble form of approximately 50 amino acids [22] (Figure 1). Human-secreted EREG is biologically active on the basis of the stimulation of DNA synthesis under the maintenance of normal cell growth [23]. The binding of soluble EREG to ERBB, such as EGFR and ERBB4, initiates the downstream signaling cascade [23]. EREG plays a critical role in the normal regulation of physiological stress, inflammation, and angiogenesis. GPCR agonists, including endothelin-1, angiotensin II, and α-thrombin, induce the cleavage of the transmembrane EREG protein, leading to the release of mature EREG that may contribute to the proliferation of vascular smooth muscle cells (VSMCs) and vascular remodeling [24]. In contrast to wild-type control cells, EREG-null macrophages released low levels of proinflammatory cytokines upon stimulation with Toll-like receptor agonists [25]. However, EREG is expressed in keratinocytes and tissue-resident macrophages where it regulates skin homeostasis, and EREG deficiency causes the development of chronic dermatitis in mice. The depletion of EREG in macrophages or dendritic cells reduces interleukin (IL)-6 production in response to peptidoglycan stimulation [26]. Cutaneous excisional wound healing and inflammation induce the expression of several genes, such as EREG and VEGF-B, that promote angiogenesis [27]. EREG expression in most human and mouse tissues is low or non-existent [28,29]. Inflammation and angiogenesis are processes that play important roles in cancer development, including the initiation of carcinogenesis, tumor in situ and advanced stages of cancer [30]. Notably, elevated EREG expression is implicated in the activation of inflammation during the early tumor initiation described in the next section.

### 2.2. EREG Promotes Early Cancer Development

The EREG gene is also overexpressed in Kirsten rat sarcoma viral oncogene homolog (KRAS)-mutant NSCLC cells or immortalized cells expressing mutant KRAS; however, the expression level is low in human noncancerous bronchial epithelial cells [31]. EREG plays a crucial role in hTERT-mediated cell immortalization and transformation of fibroblasts [32]. The physiological action of EREG-mediated ERBB2 signaling could control epithelial cell differentiation when human airway epithelial cells were cocultured with EREG-expressing fibroblasts [33]. Moreover, compressive stress induced EREG expression in human bronchial epithelial cells, and the downstream signaling pathways of EREG-mediated ERBB were repressed by an EGFR inhibitor [34]. Immunohistochemical (IHC) analysis revealed a high expression (91.7%) of the EREG protein in thymic carcinomas, indicating that EREG is commonly expressed in thymic cancer tissues [35]. Expression and distribution of aggregated EREG protein examined through IHC staining may be used as early indicators of ovarian cancer development [36]. EREG is required for normal fibroblast transformation and can induce epithelial–mesenchymal transition (EMT) through JAK2/STAT3 and IL-6 signaling pathways in oral squamous cell carcinoma (OSCC) cells [37]. Compared with the normal breast epithelium, EREG was induced in ductal carcinoma in situ (DCIS) lesions, and EREG and MMP-1 were correlated in a subset of DCIS samples [38]. Furthermore, EREG and MMP-1 overexpression increase tumor cell survival in early-stage breast cancer. The oncogenic role of long intergenic non-protein coding RNA 885 (LINC00885) is correlated with early breast cancer progression; which may promote cell proliferation and survival through EREG/EGFR and FOXM1 pathways [39].

Long-term exposure to air pollutants was significantly associated with worsening lung function and lung cancer progression [40]. 2,3,7,8-Tetrachlorodibenzo-p-dioxin, an environmental pollutant, induces EREG expression through the binding of the activated aryl hydrocarbon receptor to the *EREG* promoter, which includes a dioxin-responsive element and three Sp1 binding sites [41]. Exposure to airborne contaminants, such as polycyclic aromatic hydrocarbons, can upregulate EREG expression through the EGFR signaling pathway, thereby promoting tumor progression. The gene expression level of *EREG* is usually low in nontumor cells, but high in various tumors, including lung tumors [31]. Depletion of EREG diminished the formation of lung tumors in a primary two-stage mouse model, and exposure to 3-methylcholanthrene and butylated hydroxytoluene caused tumor initiation and progression, respectively [42]. At the early time point (12 weeks), inflammation (macrophages, polymorphonuclear leukocytes, and CXCL1/KC levels) was significantly reduced in the *EREG*-knockout (*EREG*^−/^^−^) mice compared with wild-type mice controls (*EREG*^+/+^). At 20 weeks, tumor development was also significantly decreased in the *EREG* knockout (*EREG*^−/^^−^) mice when compared with wild-type control. These findings indicate that EREG plays a critical role in the cancer initiation and cancer progression. Several carcinogens and their metabolites continue to induce activation of the oncogene (such as KRAS) or blockade of the tumor suppressor gene (such as p53) that eventually causes lung cancer progression [43]. Our recent findings revealed that a potential carcinogen, 3-nitrobenzanthrone (3-NBA), induced the downregulation of p53 protein and the transformation of lung epithelial cells. EREG overexpression markedly led to tumorigenesis in 3-NBA-transformed cells through the activation of PI3K/AKT and MEK/ERK pathways [44]. Increased levels of EREG activate EGFR downstream pathways and synergistically enhance IL-6/STAT3 signaling in malignant tumor progression [45]. Apical EREG maintains the activation of EGFR phosphorylation and downstream signaling pathways; however, basolateral EREG stimulation only leads to transient EGFR tyrosine phosphorylation. Moreover, the apical mistrafficking of EREG (Y156A) markedly leads to larger, highly proliferative, and aggressive tumors. EREG deficiency in mice promoted dextran sulfate sodium—induced intestinal damage and did not alter intestinal tumor development [46]. The action of stromal-derived EREG prevents intestinal damage and causes colitis-associated cancer [46,47]. In addition, Yap-dependent EGFR activation signaling drives cancer initiation in the intestine, thus contributing to regeneration and tumorigenesis [48]. In the gland epithelial development microenvironment, the nuclear Yap promotes the expression of EREG and consequently activates the EGFR signal pathway [49]. Atypically high concentrations of EREG, but not of other EGFR ligands (such as EGF and AREG), could rescue Yap-null organoid growth [48]. The findings suggest that increased stromal EREG compensates for the regeneration function due to the loss of Yap *in vivo*. Future longitudinal studies should examine the potential carcinogenic characteristics of environmental contaminants to understand the implications of EREG-mediated tumor initiation. Moreover, a comprehensive understanding of pathways obtained through multiomic analyses may clarify the detailed characteristics of TMEs and the spatial compartmentalization of EREG/EGFR signaling and crucial related molecules, thus providing new insights into treatment against cancer development.

## 3. The Expression Levels of EREG during Cancer Progression

### 3.1. Elevated Expressions of EREG in Various Cancer Types

The activated EREG/EGFR pathway regulates various cellular functions, including cancer cell proliferation, survival, metastasis, and angiogenesis, thereby conferring malignant tumor phenotypes [50]. Clinicopathological and in vitro studies have revealed that EREG expression has prognostic importance in several human cancers. Table 1 summarizes the potential functions of EREG. EREG is usually overexpressed in various human cancers such as bladder cancer [51,52,53], brain cancer [54,55,56,57], breast cancer [38,39,58,59,60,61,62,63], ovarian and cervical cancer [36,64], colorectal cancer [47,65,66,67,68,69,70,71,72], head and neck cancer [11,37,73,74,75,76], liver cancer [77,78], lung cancer [31,79,80,81,82], pancreatic cancer [83], prostate cancer [84], gastric cancer (GC) [85,86], and thymic cancer [35].

IHC staining analysis revealed that EREG expression was observed in 237 (64.7%) of 366 biopsy tissues obtained from patients with NSCLC and was correlated with nodal metastasis and shorter survival [80]. EREG proteins were significantly overexpressed in lung adenocarcinomas and correlated with aggressive tumor phenotypes [31]. A pan-cancer survival analysis of cancer hallmark genes in cervical cancer indicated that EREG can cause replicative immortality [64]. In bladder cancer, *EREG* expression was increased in patients with the advanced disease stage, and high *EREG* mRNA expression was substantially associated with poor survival outcomes [51]. The EREG protein level was increased in GC tissues and correlated with the tumor-node-metastasis stage and shorter overall survival (OS) [86]. In addition, increased *EREG* mRNA expression was associated with short OS in patients with OSCC [73]. The EREG protein level in CRC tissues was significantly associated with invasion and distant tumor metastasis [65]. In addition, high expression of *EREG* mRNA, but not *AREG,* was correlated with longer OS and progression-free survival (PFS) in patients with mCRC receiving first-line irinotecan-based chemotherapy [68]. Moreover, EREG can serve as a potential predictive marker, and high EREG protein levels were associated with better survival outcomes in patients with CRC receiving neoadjuvant concurrent chemoradiotherapy [71]. Moreover, increased EREG protein expression in patients with glioblastoma was highly correlated with a higher tumor grade and poor OS [55]. Although a high EREG protein level was found in GC tissues and associated with poor outcomes [86], the low *EREG* expression could be caused by epigenetic regulatory mechanisms, such as aberrant histone modification and DNA methylation, in a subset of patients with GC [85].

### 3.2. The Outcomes of EREG Expression and KRAS Mutation

The survival outcomes of patients with lung cancer are substantially related to EGFR mutations (such as L858R and exon 19 deletion or insertion) or overexpressed ERBB family members that promote EGFR activity by increasing dimerization or ATP affinities [87,88]. The specific inhibition of EGFR through treatment with tyrosine kinase inhibitors (TKIs), such as gefitinib and erlotinib, initially showed satisfactory clinical outcomes [89]. Although patients with metastatic colorectal cancer (mCRC) exhibited a high efficacy to anti-EGFR antibodies such as cetuximab or panitumumab, drug resistance was also observed in cancer cells [90,91]. Most patients with CRC who undergo anti-EGFR therapy exhibit an increased EGFR copy number; however, the degree of EGFR expression does not seem to correlate with the blockade of EGFR signaling. The increased EGFR gene copy number is mostly associated with a better outcome of anti-EGFR monoclonal antibodies treatment, particularly among those patients with wild-type KRAS [92]. However, in KRAS-mutated patients, the difference often did not exist. The compensation of alternative signaling pathways in various oncogenic mutations and different cell contexts, including aberrant EREG expression, may lead to the unfavorable outcomes of anti-EGFR therapies in various cancer cells [91,93].

**Figure 2:**
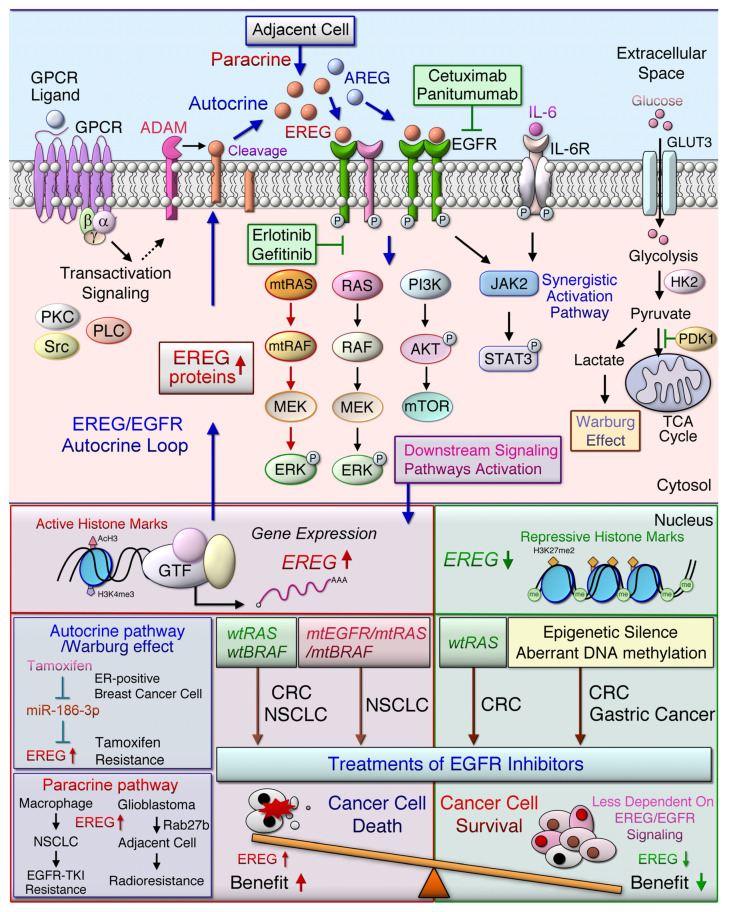
The EGFR/ERBB signaling pathway mediated by EREG leads to the cancer development and distinct drug response. G-protein-coupled receptor (GPCR) activation induces the cleavage of transmembrane epiregulin (EREG) protein and then secretes mature EREG. Soluble EREG binds to ERBB, such as epidermal growth factor receptor (EGFR) and ERBB4, which initiate the downstream signaling cascade, whereas the ligand protein is cleaved by a disintegrin and metalloproteinase enzyme (ADAM). The homodimerized or heterodimerized ERBB activate RAS (rat sarcoma)/RAF (rapidly accelerated fibrosarcoma) and phosphatidylinositol-4,5-bisphosphate 3-kinase (PI3K)/AKT (a serine/threonine protein kinase) signaling cascades and synergistically activate, signal transducer and activator of transcription (STAT) 3 signaling pathways, which then induced the upregulation of *EREG* downstream signaling pathways. Oncogenic mutations in EGFR, KRAS, or BRAF genes in non-small cell lung cancer (NSCLC) cells lead to the constitutive activation of the downstream signaling, which in turn upregulates *EREG* expression. Treatment with anti-EGFR antibodies, such as cetuximab or panitumumab, in patients with metastatic colorectal cancer (mCRC) with wild-type RAS improved patient outcomes. EREG overexpression was found in wild-type, mutant EGFR (mtEGFR), or mutant BRAF (mtBRAF) NSCLC cells that are sensitive to anti-EREG antibodies or an EGFR-tyrosine kinase inhibitor (EGFR-TKI, gefitinib or erlotinib). EREG might diminish TKI-induced NSCLC cell apoptosis through EGFR/ERBB2 and AKT signaling pathways. However, the low-level expression of AREG and EREG in CRC cells indicates that tumors are less dependent on EGFR, which is particularly prone to cause EGFR inhibitors resistance. Aberrant genetic alterations, including mutant RAS (mtRAS) and mtBRAF in CRC, induce resistance to anti-EGFR therapy. Low EREG expression was caused by aberrant histone modification and DNA methylation in a subset of cancer patients, such as those with gastric cancer, which cause resistance to anti-EGFR therapy. The miR-186-3p/EREG axis as a key regulatory pathway can induce the Warburg effect through EGFR signal activation, thereby increasing the expression of glycolytic genes, including glucose transporter 3 (GLUT3), hexokinase 2 (HK2), and pyruvate dehydrogenase kinase 1 (PDK1) in breast cancer cells resistant to tamoxifen. In addition, Rab27b mediates radioresistance in highly malignant glioblastoma (GBM) cells through the EREG-mediated paracrine pathway.

In NSCLC cells, EREG acts as an ERBB ligand and a potential transcription target of oncogenic KRAS signaling [31]. The oncogenic activation of the MEK (mitogen-activated protein kinase)/ERK (extracellular signal-regulated kinase) signaling pathway by mutant EGFR, KRAS, or BRAF genes may induce EREG overexpression [79]. When the number of EGFR ligands is increased, these ligands can bind to EGFR/ERBB and activate downstream signaling pathways, including MEK/ERK and PI3K/AKT, through an autocrine loop mechanism (Figure 2). Patients with NSCLC exhibiting high EREG expression and KRAS mutations had shorter OS and disease-free survival than did patients with low EREG expression and wild-type KRAS [79]. In addition, *EREG* mRNA levels are higher in pancreatic ductal adenocarcinoma (PDA) tissues than in normal and chronic pancreatitis tissues [83]. The findings of whole-exome sequencing analysis also indicated that *EREG* expression is induced in oncogenic KRAS-driven PDAs [94]. Notably, the oncogenic driver mutations, such as EGFR and KRAS, are commonly observed in lung adenocarcinoma [95]. Moreover, tumor-promoting functions were blocked by anti-EREG antibodies or an EGFR-tyrosine kinase inhibitor (EGFR-TKI, gefitinib or erlotinib) in NSCLC [80]. Thus, it is regarded as likely that a subset of NSCLC patients with high EREG expression and driver mutation are beneficial for anti-EGFR or targeting EREG treatments.

Monoclonal antibodies, such as cetuximab and panitumumab, target the extracellular domain of EGFR to block ligand binding and intracellular signaling, thus inhibiting tumor cell proliferation, angiogenesis, and metastasis [89]. Cetuximab and panitumumab have been approved for advanced CRC and late-stage head and neck carcinoma (HNC), and metastatic CRC, respectively [91]. The benefit of cetuximab or panitumumab monotherapy alone or in combination with chemotherapy is limited only to patients with mCRC with wild-type KRAS and NRAS (neuroblastoma RAS viral oncogene homolog) [96,97,98,99,100,101]; however, the use of anti-EGFR antibodies in patients with constitutively activated RAS had poor treatment outcomes [93]. AREG and EREG ligands are usually highly expressed in CRC and activate EGFR downstream pathways [65,98]. AREG and EREG activated the downstream EGFR-RAS-MAPK signaling axis, which is the positive autocrine loop that was anticipated as a regulation mechanism [102]. In a previous meta-analysis, patients with mCRC with wild-type RAS, as well as AREG and EREG overexpression, treated with cetuximab or panitumumab exhibited better survival outcomes than others [103]. Increased *AREG* or *EREG* gene expression is correlated with the benefits of anti-EGFR therapies in nonrandomized patients with advanced CRC (aCRC) and wild-type KRAS [97,104,105]. In a phase III trial study (CO.17), patients with aCRC with wild-type *KRAS* and high *EREG* mRNA expression, but not low *EREG* expression, exhibited prolonged OS and PFS after they received cetuximab therapy [106].

The therapeutic efficacy of panitumumab alone and in combination with FOLFOX4 chemotherapy that includes oxaliplatin, fluorouracil (FU), and leucovorin in patients with aCRC with RAS mutations was poorer than that in patients with wild-type RAS [100]. These results suggest that patients with CRC with elevated EGFR signaling would be more sensitive to the anti-EGFR therapy. However, patients with aCRC with wild-type RAS showed no improvement in OS when treated with a combinational therapy of irinotecan and panitumumab [101]. Nonetheless, a large randomized clinical trial (PICCOLO) showed that the high expression of EGFR ligands, such as AREG and EREG, is predictive of a prolonged PFS in patients with aCRC with wild-type RAS receiving panitumumab [96]. In addition, high HER3 and *AREG*/*EREG* mRNA expression in patients with aCRC, with wild-type *RAS* receiving panitumumab, was associated with longer PFS and OS [107]. Patients with mCRC, with wild-type RAS who had left-sided tumors and were receiving cetuximab, had prolonged PFS, and more than half (150/399) of right-sided tumors in these patients had *KRAS* or *PIK3CA* (PI3K, catalytic subunit alpha) mutations [108]. *EREG* and *AREG* gene expression was markedly limited by DNA methylation, which is associated with CpG island methylator phenotype status in patients with primary wild-type *KRAS* tumors that may be resistant to therapeutic anti-EGFR antibodies [109]. Both *AREG* and *EREG* gene expression are negatively regulated by complex mechanisms involving intragene methylation and promoter-dependent control [102]. *AREG* and *EREG* expression was inversely correlated with methylation levels and the elevated status of the CpG island methylator phenotype. Besides, the poorer PFS with anti-EGFR therapy was associated with the CpG island methylator phenotype-high status [109].

### 3.3. Alternative Signaling Pathways

**Figure 3:**
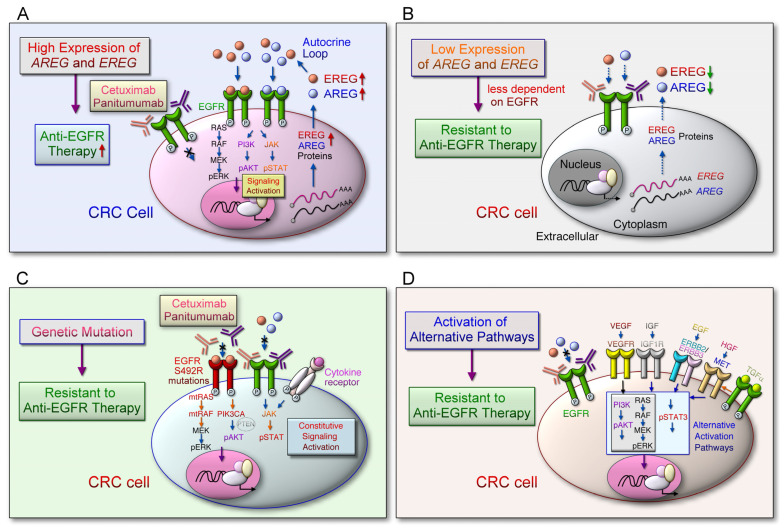
The alternative pathways and mechanisms bypass targeting EREG-mediated EGFR signal activation in colorectal cancer cells. (**A**) The expression of AREG and EREG are coordinately regulated by an autocrine loop through EGFR downstream signaling activation, which plays an important role in tumor growth and survival. The EGFR ligand binds to the EGFR and causes downstream signaling pathways that are essential for cell growth and proliferation. Cetuximab or panitumumab prevents the ligand from binding to EGFR, thereby blocking EGFR signaling. (**B**) Low *AREG* and *EREG* gene expression levels are associated with resistance to anti-EGFR therapy. The low expression levels of AREG and EREG indicate that tumor progression is less dependent on EGFR activation; therefore, the cancer cells are particularly prone to less response to EGFR inhibitor treatment. (**C**) Aberrant genetic alterations, including RAS, BRAF, PIK3CA, EGFR S492R mutations, PTEN loss, and STAT3 phosphorylation in the EGFR signaling pathways induce resistance to anti-EGFR therapy. These constitutively activate the downstream signal cascade of EGFR leading to resistance to anti-EGFR therapy, regardless of EGFR blockade. (**D**) Aberrant activation of the alternative pathways can induce resistance to anti-EGFR therapy. EGFR downstream effectors can be activated by activating compensatory membrane growth factor receptors, including IGF-1R, MET, HER2 and VEGFR. The stimulation of the corresponding growth factors causes the intracellular signaling pathway to bypass EGFR and induce tumor cell growth and proliferation, leading to resistance to anti-EGFR therapy.

Low *AREG* and *EREG* mRNA levels in mCRC tumor tissues are associated with BRAF mutations and correlated with shorter OS in patients with cancer-receiving oxaliplatin/fluoropyrimidine and bevacizumab as combinational treatment [69]. The expression levels of AREG and EREG ligands are coordinately regulated, and EGFR downstream signaling pathways can be activated by the autocrine/paracrine ligand loop to promote cancer progression (Figure 3A). The low-level expression of *AREG* and *EREG* indicate that tumors are less dependent on EGFR; thus, it is particularly prone to drug resistance to EGFR inhibitors (Figure 3B). However, in certain tumors (such as CRC) with aberrant genetic alterations, including RAS [110], BRAF [69], PIK3CA [111], EGFR S492R mutations [112], PTEN loss [113], and STAT3 phosphorylation [114] confer insensitivity to anti-EGFR therapy through constitutive activation of EGFR downstream signaling cascades regardless of EGFR blockade (Figure 3C). Additionally, EGFR downstream pathways can be activated by compensatory activation of growth factor receptors, including insulin-like growth factor 1 receptor (IGF-1R) [115], MET (MET proto-oncogene, receptor tyrosine kinase) [116], ERBB2 [117], and VEGFR [118] (Figure 3D). These alternative pathways may trigger intracellular signaling pathways to bypass EGFR and induce tumor cell growth and proliferation, leading to resistance to anti-EGFR therapies. High AREG and EREG expression levels in patients with mCRC with wild-type RAS could indicate the better efficacy of anti-EGFR therapies [103]. Furthermore, BRAF mutation, accompanied by low *AREG* and *EREG* mRNA expression levels, was correlated with poor survival outcomes in patients with mCRC treated with oxaliplatin/fluoropyrimidine and bevacizumab [69].

Notably, the methylation of the *AREG* gene is particularly diminished in the epithelial compartment of the CRC TME compared with in stromal tissue and normal epithelial cells [102]. Compared with radiotherapy alone, cetuximab and radiotherapy combined therapy resulted in prolonged PFS in patients with advanced head and neck squamous cell carcinoma (HNSCC) [119]. Moreover, patients with recurrent/metastatic HNSCC receiving combined treatment of cetuximab and chemotherapy, and demonstrating high AREG and EREG expression, had longer PFS and OS than those with lower expression [74]. Remarkably, high expression of EREG was suggested to fuel an oncogenic feedback loop that activates the EGFR/ERBB4 signaling cascade and was anticipated to be a therapeutic target in NSCLC [79]. Elevation of EREG has been shown to be a predictive biomarker of response to anti-EGFR therapies in mCRC and HNSCC patients [11,120]. Thus, the EGFR-specific ligands, such as EREG, have a significant effect on intracellular pathway and are strongly associated with response to anti-EGFR therapy. Understanding how specific tumor-promoting factors such as EREG are regulated under different cellular contexts and how oncogenic mutations confer alternative signal activation can help clinicians improve treatments, such as anti-EGFR therapy.

## 4. Actions of EREG in the TME

### 4.1. EREG Promotes Tumorigenicity

**Figure 4:**
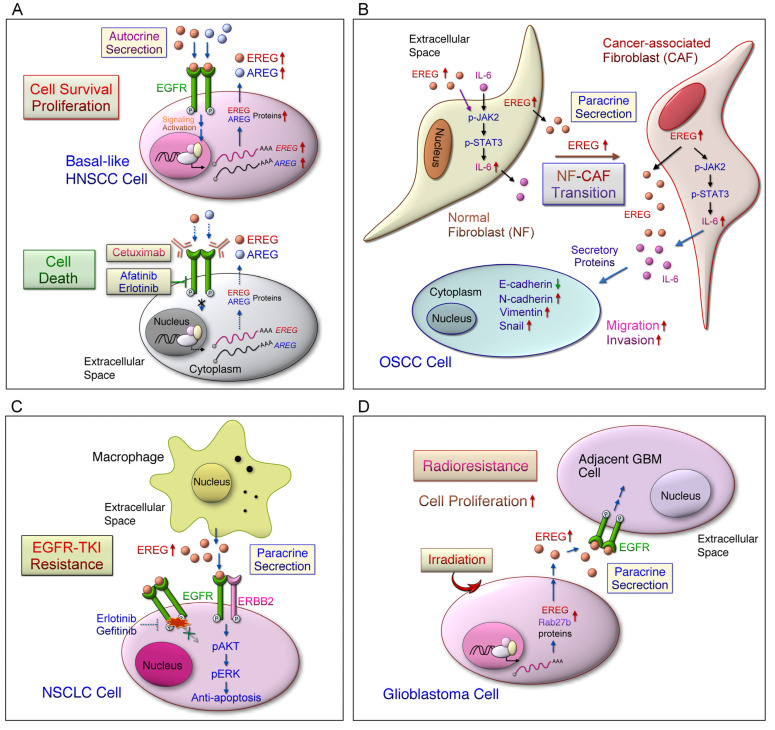
Elevated EREG expression in certain cell types may alter tumorigenesis and therapeutic response in the tumor microenvironment. (**A**) Inter-tumor heterogeneity may hinder the therapeutic efficiency of anti-EGFR treatments in head and neck squamous cell carcinomas (HNSCC). This may be caused by the dysregulated expression of factors, such as EREG, involved in the EGFR signaling pathway. Notably, basal-like cell lines are more sensitive to EGFR blockade alone or in combination with treatments targeting MEK, mTOR, or ERBB2. Additionally, EREG expression may be a predictive functional marker of anti-EGFR therapy in basal-like HNSCC. (**B**) The local resident normal fibroblasts (NFs) are converted to cancer-associated fibroblasts (CAFs) in oral squamous cell carcinoma (OSCC), which exhibit tumor-supportive properties. EREG is the most remarkably upregulated gene in CAFs. Overexpression of EREG in NFs activated the CAF phenotype. Mechanistically, the JAK2/STAT3 pathway was enhanced by EREG in parallel with increased IL-6 expression. IL-6 induced the JAK2/STAT3/EREG pathway in a feedback loop. Moreover, EREG-induced CAF activation promotes the epithelial-mesenchymal transition (EMT) necessary for migration and invasion, which depends on JAK2/STAT3 signaling and IL-6. (**C**) Among EGFR ligands, EREG significantly reduces the sensitivity of cells to EGFR TKI, which may be correlated with the resistance to erlotinib in NSCLC patients. EREG induces AKT phosphorylation in an ERBB2-dependent manner and attenuates TKI-induced apoptosis. Regardless of treatment, EREG induces the formation of EGFR/ERBB2 heterodimers. However, overexpression or knockdown of EREG in cancer cells has little effect on TKI sensitivity. EREG-rich macrophage conditioned medium induces EGFR-TKI resistance. (**D**) Rab27b mediates radioresistance in highly malignant glioblastoma (GBM) cells. In addition, Rab27b promotes the proliferation of neighboring cells through EREG-mediated paracrine signals after irradiation.

ERBB ligand EREG-mediated tumorigenesis and downstream signaling cascade under distinct genetic backgrounds has largely been suggested in cell, animal, and clinical studies (Figure 2 and Figure 3 and Table 1). In a COX2-overexpression mouse model, EREG was the most highly expressed growth factor in bladder carcinoma and promoted cancer cell proliferation [53]. Dual knockdown of EREG and N-RAS induces cell cycle arrest and suppresses liver cancer cell growth through AKT, ERK, and retinoblastoma protein (Rb) pathways [77]. Salivary adenoid cystic carcinoma (SACC) cells showed high EREG expression that promoted migration and invasion through activated AKT and ERK signaling pathways [75]. EGFRs were constitutively activated by autocrine EREG expression in SACC cells that conferred metastatic ability through downstream AKT/ERK and STAT3 signaling pathways and snail/slug protein stabilization [76]. AREG and EREG mediate the activation of the EREG downstream signaling pathway, and the overexpression of both ligands promoted basal cell clonogenic survival, which was blocked by cetuximab in basal-like HNSCC (Figure 4A) [121]. EREG activated EGFR–ERK signaling pathway and induced C-Myc expression, thus promoting oncogenic transformation in patients with HNSCC and increasing sensitivity to erlotinib [11]. Cytoplasmic EREG accumulating in ovarian cancer tissues may act through autocrine and paracrine release and bind to EGFR in the TME [36]. EREG derived from fibroblasts promotes the proliferation of intestinal epithelial cells through the ERK pathway in colitis-associated tumor development [47]. Depletion of MUC1 deficiency in fibroblasts and epithelial cells led to increased EREG expression that promoted lung cancer development through the EGFR/AKT pathway [81]. This finding suggested that the tumor-promoting role of MUC1 is compensated by increased EREG production in the TME. Depletion of tumor suppressor gene-Indian hedgehog increased EREG/*Adenoma Polyposis Coli* (Apc) pathway-driven intestinal epithelial transformation in colonic stromal cells [72]. Collectively, the results indicate that autocrine and paracrine EREG may mainly activate EGFR downstream pathways in various cancer TMEs that contribute to tumorigenesis (Figure 4).

### 4.2. EREG Mediates Tumor Metastasis

EREG promotes tumor progression and metastasis in various cancers. EREG is overexpressed in bladder cancer, which leads to a high risk of lung metastasis [52]. A set of lung metastasis signature (LMS) genes, including EREG, COX2, and MMP1/2, was identified in breast cancer cells with lung metastasis potential [59]. LMS genes mediate primary tumor growth, angiogenesis, and metastatic extravasation in breast cancer [60]. Moreover, the blockade of these mediators by combination drugs (cetuximab/celecoxib/GM6001) significantly reduced metastatic progression. KAP1 overexpression activates EREG, COX2, and MMPs, that stimulated tumor cell proliferation [61]. K14 is highly expressed in breast epithelial tumor cell clusters that are a key regulator of distant organ metastasis through the activation of EREG signaling [62]. In colon cancers with liver metastasis, EREG was identified as a metastasis-associated gene through gene expression analysis [66]. In addition, up-regulation of EREG/ERK/AKT signaling in SACC cells increased the potential of lung metastasis [75]. EREG overexpression in normal fibroblasts mediated the cancer-associated phenotype, which promoted EMT through JAK2/STAT3 and IL-6 signaling pathways [37] (Figure 4B). The findings suggest that EREG is required for fibroblast transformation in OSCC progression and that EREG-mediated OSCC migration and invasion can be therapeutically targeted in the TME. Anti-EREG antibody significantly repressed cell adhesion and spread in EREG-expressing colon cells, but exerted a slight effect on their growth [122]. Moreover, the anti-EREG antibody efficiently inhibited downstream EGFR signaling activated by EREG, but not by EGF. These findings indicated that EREG–EGFR signaling is associated with cell adhesion and migration.

### 4.3. EREG Expression Correlates with Cancer Stem Cell Characteristics

EREG expression was increased in CRC cells and was associated with cancer stem cell (CSC) characteristics [123]. In lung adenocarcinoma, LGR5 expression was examined through IHC staining, and its expression was significantly correlated with large tumor size, TNM stage, and poor prognosis [124]. LGR5-positive cells with CSC properties with increased cell proliferation ability and were converted to LGR5-negative cells with drug resistance state when exposure to chemotherapy drugs treatment. EREG protein expression levels were both detected in LGR5-positive and drug-resistant LGR5-negative colon cancer cells. Besides, the anti-EREG antibody exhibited antitumor activity against tumors derived from the LGR5-positive and LGR5-negative cells in a metastatic model. This is the first demonstration of the establishment of stable cell lines having CSC properties and the ability to transition between the two distinct states, a proliferating and a drug-resistant state. In addition, treatment with anti-EREG antibodies could effectively combat tumor metastasis when CSCs are abundant in the early stages of cancer development, indicating that targeting EREG may be an option for CSC and drug resistance therapy. Analysis of the epigenetically regulated mRNA expression-based stemness index (mRNAsi) in The Cancer Genome Atlas data set revealed that a higher EREG-mRNAsi score was correlated with shorter OS in patients with glioma [56]. Collectively, EREG overexpression results in CSC properties and plays a critical role in metastasis during tumor progression. EREG expression is potentially induced in colon CSCs and associated with drug resistance. EREG is usually overexpressed in various cancers, including glioma and lung cancer; however, whether the expression of EREG plays a critical role in distinct types of cancers with CSC properties that confer tumor metastasis and drug resistance remains unclear.

### 4.4. EREG Mediates Drug Resistance

EGFR-TKI, gefitinib, and targeted therapy in Asian patients with NSCLC with EGFR mutations yielded more favorable outcomes in terms of the objective response rate and median PFS relative to carboplatin/paclitaxel chemotherapy [125]. However, acquired resistance within 9–14 months still occurred, although EGFR-TKI treatment demonstrated an initial improvement in clinical outcomes [125,126]. Thus, studies should examine how to overcome EGFR-TKI resistance in cancer therapies. A recent study revealed that the EREG ligand causes TKI (such as erlotinib) resistance in patients with NSCLC [82] (Figure 4C). EREG reduced TKI-induced cellular apoptosis through EGFR/ERBB2 and AKT signaling pathways. However, no significant difference was noted in TKI resistance after EREG overexpression or knockdowns. EREG was mainly expressed in macrophages in the NSCLC TME, as observed through single-cell RNA sequencing [82]. Notably, EGFR-TKI resistance increased after treatment with conditional medium obtained from EREG-enriched macrophages. EREG produced by tumor-associated macrophages (TAMs) causes NSCLC cell drug resistance in the TME; however, the interplay of critical factors such as EREG expression in various TMEs in terms of different space, time, boundaries, cell types, and contexts remains unclear.

EREG can induce the Warburg effect through EGFR signaling activation, which increases the expression of glycolytic genes, including GLUT3, HK2, and PDK1, in breast cancer cells resistant to tamoxifen [63] (Figure 2). This study revealed that tamoxifen enhanced EREG expression by suppressing the inhibition of miR-186-3p targeting EREG in drug-resistant cells. In patients with CRC, EREG is a major obstacle of 5-FU treatment, and it can cause acquired resistance [70]. Hydrogen sulfide (H2S) induced thymidylate synthetase (TYMS) and EREG gene expression through the downregulation of miR-215-5p in CRC cells [70]. Furthermore, the inhibition of H2S synthesis could suppress the acquired resistance to 5-FU by regulating the miR-215-5p/EREG/TYMS axis. EREG is highly expressed in glioblastoma and can activate ERK singling to promote tumorigenesis [55]. The Rab27b protein level was specifically induced in irradiated human U87MG glioblastoma cells [57] (Figure 4D). In addition, the Rab27b-mediated production of EREG secreted from U87MG cells activated EGFR, which promoted the proliferation of H4 glioma cells in a paracrine manner. The Rab27b-EREG pathway may improve the efficacy of radiotherapy for glioblastoma multiforme (GBM). Therefore, autocrine and paracrine EREG overexpression in distinct cells (Figure 2 and Figure 3) may synergistically activate different signaling cascades, miRNA, and aerobic glycolysis pathways that promote primary tumor growth, metastasis, and drug resistance in the TME.

Drug resistance caused by oncogene mutations or activation of oncogenic signaling pathways and increased tumor plasticity is the major obstacle encountered in the application of target therapies such as anti-EGFR therapy [91,127]. Coexisting genetic events, including driver mutations and alternative signaling activation, such as EREG expression, may create distinct TMEs, leading to differential drug resistance in different tumor types. Thus, tailored therapies involving the use of combinational drugs might overcome adverse effects associated with the malignant tumor phenotype and acquired resistance.

## 5. Potential Therapeutic Targeting of EREG/EGFR and Alternative Pathways

### 5.1. Targeting the EREG/EGFR Pathway

**Figure 5:**
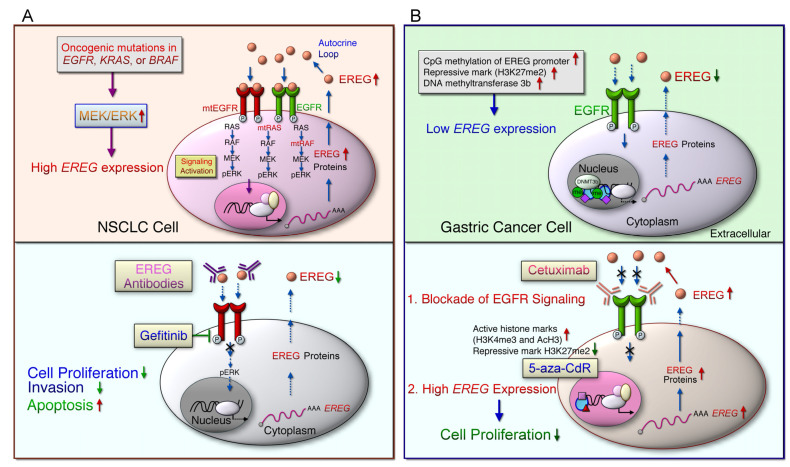
Potential targeting of EREG/EGFR may be applied to a subset of NSCLC and gastric cancer patients. (**A**) High *EREG* expression levels were found in the EGFR-mutant, BRAF-mutant NSCLC cells, a subset of NSCLC cells with wild-type EGFR/KRAS/BRAF. In NSCLC cells overexpressing EREG, the inhibition of MEK or ERK could reduce the expression of EREG regardless of the mutation status. Therefore, the activation of the MEK/ERK pathway is a common mechanism of *EREG* upregulation in NSCLC. *EREG* levels are decreased by siRNA-mediated *EGFR* knockdown and EGFR inhibitors in EGFR-mutant NSCLC cells. Moreover, lung tumors of mutant EGFR transgenic mice exhibit high *EREG* expression. In NSCLC cells with EGFR mutations, both EREG knockdown and anti-EREG antibodies inhibit cell proliferation and invasion and induce apoptosis. Collectively, targeting EREG may be a therapeutic option for EGFR-mutant NSCLC cells with resistance to EGFR-TKIs. (**B**) *EREG* is epigenetically silenced in gastric cancer cells through aberrant DNA methylation and histone modification. *EREG* is methylated and reduced in human gastric cancer cells and primary tissues from a subset of gastric cancer patients. *EREG* gene expression was reduced by aberrant CpG methylation of the *EREG* promoter. In addition, treatment with 5-aza-CdR demethylated the CpG site in the *EREG* promoter, which resulted in the rescue of *EREG* expression. DNA methyltransferase 3 beta (DNMT3b) predominantly regulates CpG methylation and silencing of the *EREG* gene. Moreover, treatment with 5-aza-CdR dynamically increased active histone marks (H3K4me3 and AcH3) and decreased the repressive mark (H3K27me2). The combination treatment with 5-aza-CdR and cetuximab exerts a synergistic antiproliferative effect on gastric cancer cells.

Treatment with the small-molecule EGFR inhibitor gefitinib suppressed EREG/EGFR downstream signaling pathways and reduced tumor formation in xenograft animals bearing EREG-overexpressing GBM cells [55]. Furthermore, treatment with the anti-EREG antibody significantly suppressed colon cancer cell adhesion and spread [122]. Compared with EGF, EREG resulted in a weaker EGFR dimer with a shorter life span, which triggered aberrant EGFR signaling and sustained ERK pathways, leading to breast cancer cell differentiation [128]. Notably, aberrant mutations of EGFR, BRAF, or KRAS occurred in lung adenocarcinomas in a mutually exclusive manner [129,130]. EREG overexpression was found in mutant EGFR or BRAF NSCLC cells and a subset of wild-type EGFR/KRAS/BRAF NSCLC cells [31,80] (Figure 5A). Knockdown of EREG suppressed anchorage-dependent or anchorage-independent cell growth in NSCLC cells with both KRAS mutations and EREG overexpression [31], indicating the therapeutic potential of targeting EREG in KRAS-driven NSCLC. Regardless of the mutation status, blockade of MEK/ERK pathways diminished EREG levels in EREG-overexpressing NSCLC cells [31]. EREG expression was suppressed by the EGFR-TKI gefitinib in EGFR-mutant NSCLC cells [80]. EGFR-mutant cell invasion was suppressed by the knockdown of EREG expression or treatment with the anti-EREG antibody. Increased expression of both AREG and EREG genes in the lung TME further promoted lung development in mutant EGFR transgenic mice [131]. Moreover, outcomes of anti-EGFR antibody treatment were improved in patients with CRC with wild-type KRAS, instead of mutant RAS, having high EREG expression [96,98,104,106]. Therefore, targeting EREG/EGFR in the TME may provide therapeutic options for patients with NSCLC, particularly a subset of NSCLC with driver mutation or resistance to EGFR-TKIs, and patients with CRC with wild-type KRAS expression.

### 5.2. Targeting the Hypermethylation of EREG

Low EREG expression is potentially regulated by aberrant DNA methylation and epigenetic modification in GC cells and tissues [85] (Figure 5B). The use of DNA demethylating agents, including 5-azacitidine (5-Aza-CR) and 5-aza-2′-deoxycytidine (5-Aza-CdR), has been permitted by the US Food and Drug Administration for treating myelodysplastic syndrome and chronic myelomonocytic leukemia. In addition, the combination treatment with 5-Aza-CdR and cetuximab synergistically exerts antiproliferation effect in GC cells [85]. Similarly, EREG gene expression is suppressed by DNA methylation in CRC cells [102,132]. An inverse correlation was observed between EREG gene expression and DNA methylation in other cancers, including head and neck, lung, and bladder cancer [132]. Elevated EREG expression resulting from the demethylation of the EREG promoter in CRC, could activate the EGFR signaling pathway [132]. Thus far, several agents targeting epigenetic regulators have been examined for solid tumors in clinical trials [133]. To prevent the risk of mutations and genome instability, nonnucleoside analogs have been developed to prevent abnormal DNA hypermethylation. Therefore, future preclinical and clinical trials should evaluate the efficacy of anti-EGFR therapy combined with demethylation drugs to suppress the inhibition of EREG gene expression and re-initiation of EREG-mediated signal pathways.

### 5.3. Targeting the Alternative Activation Signaling Pathway

Patients with CRC with wild-type KRAS and high EREG gene expression appeared to benefit from anti-EGFR therapy [98,104,106]. However, other unidentified molecular determinants of response to anti-EGFR therapy should be identified. The constitutive activation of EGFR signaling pathways caused by several oncogenic mutations, such as KRAS, NRAS, BRAF, and PIK3CA, contribute to drug resistance [134]. Although not all patients with KRAS mutations develop EGFR resistance, more than 80% of those patients have KRAS codons 12 and 13 mutations [135]. Sotorasib (AMG-510) is a selective and irreversible KRAS^G12C^ inhibitor that restrains KRAS in the inactive GDP-bound state. The effects of specific KRAS inhibitors, such as AMG-510 and MRTX849 targeting specific G12C mutations (KRAS^G12C^) are being investigated in ongoing clinical trials in non-small cell lung cancer [136]. In a clinical trial study evaluating the effect of sotorasib, patients with lung cancer exhibited a better tumor response than did patients with CRC [137]. The inconsistency may result from other critical pathways, such as Wnt or EGFR signaling, suggesting that KRAS inhibition alone is not sufficient to suppress CRC progression [138,139,140]. EGFR signaling is the main mechanism in CRC resistance to KRAS^G12C^ inhibitors, that is, the combined blockade of EGFR and KRAS^G12C^ signaling can effectively inhibit tumors in CRC-derived organoids and xenografts [138]. However, studies should examine the correlation of EREG expression in the combined therapeutic strategy of EGFR and KRAS inhibitors to treat patients with CRC with KRAS^G12C^. In addition, low AREG and EREG protein levels were correlated with BRAF mutations in patients with mCRC [69]. Cancer cells with low expression of AREG and EREG may possibly indicated that it not sensitive to anti-EGFR therapies. The combination treatments of BRAF inhibitors (vemurafenib) and EGFR inhibitors, namely gefitinib, erlotinib, or cetuximab, in patients with CRC synergistically inhibited mutated BRAF V600E (BRAF^V600E^) tumor growth in xenograft models [141]. Mechanistically, BRAF^V600E^ inhibition caused a rapid feedback activation of EGFR. A subsequent trial study showed that the combined use of the inhibitors of BRAF (dabrafenib), EGFR (panitumumab), and MEK (trametinib) provided treatment benefits in patients with BRAF^V600E^ CRC [142]. In the BEACON trial, a triplet regimen of encorafenib (a BRAF inhibitor), binimetinib (a MEK inhibitor), and cetuximab resulted in prolonged OS and better efficacy in patients with mCRC with BRAF^V600E^ compared with standard therapy [143]. Nevertheless, whether EREG expression plays a crucial role in the combined treatments of BRAF inhibition and anti-EGFR therapy should be examined. Another compensatory effect of alternative pathway activation, such as PI3K (phosphatidylinositol-4,5-bisphosphate 3-kinase) mutation, may contribute to the constitutive activation of downstream pathways in patients with CRC with acquired anti-EGFR resistance [111]. Combined treatment with a STAT3 inhibitor (cucurbitacin B) and EGFR-TKI (gefitinib) significantly induced cell apoptosis through EGFR and STAT3 pathways in CRC cells [144]. Notably, EREG also potentially mediates the activation of the STAT3 signaling pathway in head and neck cancer [37,76]. Collectively, the findings suggest that studies should examine whether the blockade of the oncogenic mutations of RAS, BRAF, or PI3K in certain types of cancer, such as CRC, restore EREG expression that mediates the activation of EGFR downstream signaling.

### 5.4. Targeting TAM in the TME

A high infiltration of TAMs is typically correlated with poor prognosis, and M1 or M2 macrophages are associated with the antitumor or protumor phenotype in response to stimuli in the TME [145]. TAMs may play tumor-promoting and immune-suppressing roles that cause tumor initiation and act as crucial immunosuppressive drivers in the TME through distinct immune cells and cancer cell interactions [146]. EGFR mutations in NSCLC are also possibly associated with an immunosuppressive status in the TME [147]. Unlike acquired resistance directly caused by oncogenic mutations in cancer cells, a recent study revealed that EREG secreted by TAMs may confer NSCLC cell drug resistance in the TME [82]. Thus, therapeutic strategies for NSCLC with EGFR-TKI resistance may be examined by the depletion of TAM or the reduction of TAM recruitment. Decreasing of TAM by lurbinectedin, an activated transcription inhibitor, had promising efficacy in preclinical studies [148]. Moreover, a preclinical study revealed that the use of anti-CCL2 antibodies or small-molecule inhibitors supports tumor suppression by targeting TAM accumulation in the TME [149]. In addition, EGFR-TKI can dynamically modulate the immunosuppressive environment in TME in a variety of ways [150]. For example, Treg suppression functions were mediated by one of the EGFR ligands AREG through the EGFR/GSK-3β/Foxp3 axis [151]. Understanding the specific role of crucial molecules such as EREG expression in cancer cells or tumor infiltrating immune cells can help in the development of targeted therapies for different tumors. This has implications for combination treatment with EGFR-TKI and altered immune status approaches related to the TME.

## 6. Conclusions

Elevated EREG expression levels appear to be a potential predictive biomarker of anti-EGFR therapies in several cancer types. The clinical efficacy of combination treatment with anti-EGFR therapy might vary with different signaling activation levels and cell contexts in the TME as well as the timing of medication, tolerable toxicity, and different subsets of patients with cancer. Additional preclinical and clinical trial studies are warranted to obtain more accurate information on oncogenic mutations, drug resistance, and altered immune status in the TME. In addition, a comprehensive strategy that involves monitoring potential biomarkers (e.g., EREG expression) to obtain genomic, proteomic, and immune profiling in tumor tissue, and obtaining liquid biopsy specimens will be helpful for providing tailored therapy. Currently, new agents are being developed for potential targets or oncogenic mutation resistance in clinical trials or being approved for markets. Therefore, determining the most favorable outcomes of combined therapies, including targeting EGFR, EREG, and other driver oncogenic genes in a subpopulation of patients with cancer is crucial.

## Figures and Tables

**Table 1:** Characterization of EREG expression and tumorigenesis in different cancers.

Cancer Type	EREG Expression	Co-Existing Genetic Background	Pathways	Tumorigenesis and Clinical Outcomes	Ref
Bladder	Up	AR, TGF-α, and HB-EGF	-	Short OS	[51]
	Up	uPA, MMP14, and TIMP-2	-	Lung Metastasis	[52]
	Up	COX2	ERK	Immune/Stress Response and Cell Cycle/Proliferation	[53]
Brain	Up	HB-EGF	EREG/EGFR autocrine loop, JNK, and under the control of IRE1α	Tumor cell growth, Migration	[54]
	Up	AEBP1	EGFR-ERK and abrogated by gefitinib	Tumor cell growth, Colony and Sphere formation, Invasion, Tumor formation, Short OS	[55]
	Up	51 differentially expressed gene (DEG) such as BUB1	Chromosome segregation and cell cycle functions in DEG	Higher EREG-mRNA stemness scores increased with glioma grade and had worse OS	[56]
	Up	Rab27b	Rab27b mediates paracrine EREG/EGFR by ionizingradiation	Tumor cell growth, Radioresistance	[57]
Breast	Up	GRO1, MMP1/2, SPARC, IL13Rα2, VCAM1, ID1, and COX2	Lung metastasis signature (LMS)	LMS extracellular proteins mediate breast cancer metastasis to lung	[59]
	Up	COX2, MMP1, and MMP2	-	Tumor growth, Angiogenesis, and Metastasis	[60]
	Up	BTC, TGFα, HB-EGF, and NRG2	ERBB/HER ligands	Tumor aggressiveness	[58]
	Up	MMP1	Partially regulated by fibroblast growth factor receptor	Tumor growth, Tumor formation of early stage breast cancer	[38]
	Up	Active SUMOlyated KAP1, COX2, MMP1/2, and CD44	KAP1 regulates multiple KRAB-ZNF	Tumor growth and Metastasis	[61]
	Up	Keratin 14 (K14)	Dependent upon K14 expression	Distant Metastasis; Metastatic niche remodeling (Tnc, AdamTs1, Jag1) and Metastasis survival (AdamTs1, Birc5)	[62]
	Up	GLUT3, HK2, and PDK1	miR-186-3p/EREG axis, EGFR/AKT/ERK	Tamoxifen resistance and Aerobic glycolysis (Warburg effect)	[63]
	Up	LINC00885	TP53, EREG/EGFR/FOXM1	Tumor cell proliferation, Migration, Invasion, and 3D growth	[39]
Cervix	Up	-	TGF-β	Replicative immortality	[64]
	Up	Epithelial cells mainly express	Frequent aggregated form occasionally in serous and mucinous tumors	The potential autocrine and paracrine effect on EGFR	[36]
Colon and Rectum	Up	AREG	-	Tumor invasion and Distant metastases	[65]
	Up	10-gene signature including AREG, COX-2, and LCK	-	Prediction of Liver metastasis	[66]
	Up	Igl-V1, Ndg1, Lgals2, and Aldh1a3	ERK	TAF-derived EREG mediated intestinal epithelial cell proliferation and tumor development	[47]
	Up	EGFR, HER2	WNT	Distal carcinomas were more often chromosome instable and patients with metastases responded to anti-EGFR therapy	[67]
	Up	Aberrant EGFR or mutant RAS- and PIK3CA expression	The prognostic effect of high EREG expression	Longer OS and DFS in mCRC patients for 5-FU/LV plus irinotecan or irinotecan plus oxaliplatin (FIRE 1-trial)	[68]
	Down	Low AREG and EREG level are associated with mutant BRAF	-	Short OS in mCRC patients for Oxaliplatin/fluoropyrimidine plus bevacizumab treatment (Phase III AIO KRK-0207 trial)	[69]
	Up	Wild-type RAS and BRAF	-	AREG and EREG expression as predictor for longer OS in the AIO KRK-0207 trial study	[69]
	Up	TYMS	miR-215-5p-EREG/TYMS axis, Decreasing H_2_S synthesis reduced EREG and TYMS expression	Inhibiting H_2_S synthesis can reverse acquired resistance to 5-Fluorouracil (5-FU)	[70]
	Up	-	-	Better outcomes of DSS, LRFS, and MeFS for neoadjuvant concurrent chemoradiotherapy	[71]
	Up	EREG, BTC, and NRG1 in gp38+ fibroblasts	Epithelium-derived Indian Hedgehog (Ihh) restricts stromal expression of ERBB ligands	Colonic adenomagenesis in APC and Ihh deficiency mice, Tumor cell proliferation and increased Lgr5+ stem cells	[72]
Head and Neck	Up	AREG	-	Longer PFS and OS in recurrent/ metastatic patients with Cetuximab and chemotherapy	[74]
	Up	C-Myc	EREG-EGFR-C-Myc, EREG mediates constitutive activation of ERGR/ERK	Tumor cell growth, Tumor formation, Sensitivity to Erlotinib, Shorter OS	[11]
	Up	HER2-4	-	OSCC Cell proliferation, Short OS	[73]
	Up	COX-2	AKT/ERK	SACC Migration, Invasion	[75]
	Up	Snail/Slug stability	EREG/EGFR, AKT/ERK and STAT3	SACC EMT, Blockade of lung metastasis by Erlotinib, Short OS	[76]
	Up	α-SMA	IL-6, JAK2/STAT3	NF-CAF transformation, CAF EMT, OSCC Tumor growth, Shorter OS.	[37]
Liver	Up	Knockdown of N-RAS	Dual knockdown reduced ERK, AKT, and Rb	Dual knockdown of N-RAS and EREG induced cell cycle arrest and inhibited cell growth	[77]
	Up	-	Intestinal microbiota and TLR4 activation	Proliferation and antiapoptosis	[78]
Lung	Up	EGFR-mutant NSCLC cells	EGFR-dependent, ERK and p38MPAK	Invasion, Proliferation, Antiapoptosis, Metastasis and Short OS	[80]
	Up	KRAS mutation	Mutant KRAS constitutive activation	Anchorage-dependent and -independent cell growth, Antiapoptosis, Short OS and DFS, Pleural involvement, Lymphatic permeation or vascular invasion	[31,79]
	Up	Muc1 deficiency in fibroblasts and malignant cells	EGFR/AKT	Cell proliferation and survival, EREG production in lung cancer Muc1-KO model, Short OS in cancer patients	[81]
	Up	Intratumoral EREG action derived from macrophages	ERBB2/AKT	EGFR-TKI resistance	[82]
	Down	87% (20/23) of SCLC cells	-	-	[31]
Pancreas	Up	-	-	Tumor cell growth, Correlated with pancreatic ductal adenocarcinoma	[83]
Prostate	Up	TGFα, AREG, HB-EGF, HER1/2	-	Expression in androgen- independent cell	[84]
Stomach	Down	Hypermethylated in gastric cancer cell (7/11, 64%) and primary tumors (4/13, 30%)	Aberrant DNA methylation and histone modification	EREG promoter methylation, 5-Aza-CdR and Cetuximab exerted a synergistic antiproliferation	[85]
	Up	-	-	Tumor size, Lymph node metastases, Distant metastases, Short OS	[86]
Thymus	Up	TGFα, HER1-3, Rare EGFR and HER2 gene amplification	-	ERBB ligand and receptor proteins commonly in primary squamous cell carcinomas	[35]

Abbreviations: AEBP1, adipocyte enhancer binding protein 1; AKT, v-akt murine thymoma viral oncogene homolog 1; Aldh1a3, aldehyde dehydrogenase family 1 member A3; α-SMA, alpha-smooth muscle actin; AREG, amphiregulin; BTC, betacellulin; Bub1, budding uninhibited by benzimidazoles 1; CAF, cancer-associated fibroblast; COX2, cyclooxygenase-2; DFS, disease-free survival; DSS, disease-specific survival; ERBB1-4, erythroblastic leukemia viral oncogene homolog; ERK, extracellular signal kinase; EREG, epiregulin; EMT, epithelial-mesenchymal transition; GLUT3, glucose transporter 3; GRO1, growth-regulated alpha protein; HB-EGF, heparin-binding EGF-like growth factor; HER 1-4, human epidermal growth factor receptor 1-4; HK2, hexokinase 2; HNSCC, head and neck squamous cell carcinoma; IL-6, Interleukin 6; IL13Rα2, interleukin 13 receptor subunit alpha 2; Igl-V1, immunoglobulin light chain V-region locus1; JAK2, Janus kinase 2; KAP1, KRAB-associated protein-1; LCK, lymphocyte-specific protein tyrosine kinase; LGALS2, galectin 2; LRFS, locoregional recurrence-free survival; mCRC, metastatic colorectal cancer; MeFS, metastasis-free survival; MMP, matrix metalloproteinase; MUC1, mucin 1; NDG1, Nur77 dependent gene-1; NRG, neuregulin; NF, normal fibroblast; NSCLC, non-small-cell lung carcinoma; OS, overall survival; OSCC, oral squamous cell carcinoma; PDK1, pyruvate dehydrogenase kinase isozyme 1; Rab27b, member RAS oncogene family; SACC, salivary adenoid cystic carcinoma; SCLC, small-cell lung carcinoma; SPARC, secreted protein acidic and cysteine rich; STAT3, signal transducer and activator of transcription 3; TAF, tumor-associated fibroblast; TGF-α, transforming growth factor alpha; TIMP, tissue inhibitor of metalloproteases; TLR4, toll like receptor 4; TYMS, thymidylate synthetase; uPA, urokinase-type plasminogen activator; VCAM1, vascular cell adhesion molecule 1; WNT, wingless (wg) and int-1.

## Data Availability

Not applicable.
